# Insect Herbivory Strongly Modifies Mountain Birch Volatile Emissions

**DOI:** 10.3389/fpls.2020.558979

**Published:** 2020-10-27

**Authors:** Jolanta Rieksta, Tao Li, Robert R. Junker, Jane U. Jepsen, Ingvild Ryde, Riikka Rinnan

**Affiliations:** ^1^Terrestrial Ecology Section, Department of Biology, University of Copenhagen, Copenhagen, Denmark; ^2^Center for Permafrost (CENPERM), Department of Geosciences and Natural Resource Management, University of Copenhagen, Copenhagen, Denmark; ^3^Evolutionary Ecology of Plants, Department of Biology, Philipps-University Marburg, Marburg, Germany; ^4^Department of Biosciences, University of Salzburg, Salzburg, Austria; ^5^Norwegian Institute for Nature Research, Fram Centre, Tromsø, Norway; ^6^Section for Plant Biochemistry, Department of Plant and Environmental Sciences, University of Copenhagen, Copenhagen, Denmark

**Keywords:** arctic, biotic stress, geometrid moth, insect herbivory, mountain birch, stress severity, volatile organic compounds

## Abstract

Insect herbivory is known to augment emissions of biogenic volatile organic compounds (BVOCs). Yet few studies have quantified BVOC responses to insect herbivory in natural populations in pan-Arctic regions. Here, we assess how quantitative and qualitative BVOC emissions change with increasing herbivore feeding intensity in the Subarctic mountain birch (*Betula pubescens* var *pumila* (L.)) forest. We conducted three field experiments in which we manipulated the larval density of geometrid moths (*Operophtera brumata* and *Epirrita autumnata*), on branches of mountain birch and measured BVOC emissions using the branch enclosure method and gas chromatography-mass spectrometry. Our study showed that herbivory significantly increased BVOC emissions from the branches damaged by larvae. BVOC emissions increased due to insect herbivory at relatively low larvae densities, causing up to 10% of leaf area loss. Insect herbivory also changed the blend composition of BVOCs, with damaged plants producing less intercorrelated BVOC blends than undamaged ones. Our results provide a quantitative understanding of the relationship between the severity of insect herbivore damage and emissions of BVOCs at larvae densities corresponding to background herbivory levels in the Subarctic mountain birch. The results have important and practical implications for modeling induced and constitutive BVOC emissions and their feedbacks to atmospheric chemistry.

## Introduction

High latitude ecosystems have experienced rapid warming (up to 1°C per decade) for the past 30 years and the temperatures in these areas continue increasing at least twice as fast as the global average (IPCC, 2014). Climate warming strongly modifies the structure and function of Arctic and Subarctic ecosystems (Callaghan et al., 2013). For example, vegetation communities are shifting in composition, shrubs are expanding to the tundra (Myers-Smith et al., 2011), and warming also influences the distribution and development of insect herbivores (Bale et al., 2002). Studies have shown that warming strongly increases emissions of biogenic volatile organic compounds (BVOCs) from Arctic vegetation (Faubert et al., 2010; Kramshøj et al., 2016; Lindwall et al., 2016), but little is known about how biotic factors such as insect herbivory, affect BVOC emissions in the Arctic and Subarctic.

Biogenic volatile organic compounds emitted by plants play diverse ecological roles, such as increasing thermotolerance (Peñuelas and Staudt, 2010), attracting pollinators and herbivore predators (Kessler and Baldwin, 2002; Dicke, 2009; Dicke and Baldwin, 2010), sealing wounds (Scala et al., 2013), and deterring insect herbivores and pathogens (Kessler and Baldwin, 2001, 2002). They also serve as semiochemicals for plant–insect and plant–plant signaling (Kessler and Baldwin, 2001, 2002; Dicke, 2009). Understanding how biotic stressors influence BVOC release to the atmosphere is also relevant from an atmospheric perspective. BVOCs undergo complex oxidation reactions in the atmosphere potentially leading to effects on the atmospheric composition, e.g., via aerosol formation and growth (Joutsensaari et al., 2015).

An increased thermal budget for growth and reproduction, as a consequence of the warmer conditions, is likely to result in larger distribution ranges for many insect species (Bale et al., 2002), which in turn, increases insect herbivore pressure on plants (Lemoine et al., 2014). Increased temperatures can act as disease vectors, because pathogens can be carried to new regions on hosts that are expanding their range (Harvell et al., 2002). In cases where host plants experience abiotic stress, such as drought, plants may exhibit increased susceptibility to diseases transmitted by herbivores (Harrington et al., 2001).

Both background herbivory and insect outbreaks are expected to increase with changing climate (Bale et al., 2002; Barrio et al., 2017) prompting further consequences on ecosystem functions. At high latitudes, insect herbivory may be the dominant factor increasing BVOC emissions during periods of active insect herbivore feeding (Li et al., 2019). Both temperature increases and higher insect herbivore pressure are likely crucial determinants of future BVOC emission rates in the high latitudes. First, temperature has a strong, direct, positive effect on BVOC emission rates, as it increases BVOC synthesis and release in Arctic plants (Ghirardo et al., 2020). Second, increased insect herbivore activity acts to further increase BVOC emission rates and the two factors (temperature and herbivory) have synergistic effects (Li et al., 2019).

A qualitative understanding of herbivore induced BVOC emissions is generally well developed (Loughrin et al., 1994; Priemé et al., 2000; Arimura et al., 2005; Schaub et al., 2010; Copolovici et al., 2011). However, quantitative relationships between herbivore damage and BVOC emissions have not been well constrained, especially under natural conditions (Arneth and Niinemets, 2010; Faiola and Taipale, 2020). Consequently, insect herbivory is not currently included in global BVOC emission models. Thus, there is a need for more quantitative, field-based studies to derive response functions between insect feeding and BVOC emissions to improve models (Faiola and Taipale, 2020).

In the Arctic and Subarctic, few studies have analyzed BVOC emissions in response to insect herbivory (Mäntylä et al., 2008; Li et al., 2019). This is a paradox given the important role insect herbivores play as drivers of ecosystem functions in high-latitude environments (Jepsen et al., 2013; Lund et al., 2017; Vindstad et al., 2019). In the mountain birch forest of Fennoscandia, geometrid moths, winter moth (*Operophtera brumata*), and autumnal moth (*Epirrita autumnata*) are known to cause severe defoliation that spans hundreds of square kilometers (Tenow, 1972; Jepsen et al., 2009). The larvae of these moth species feed mainly on mountain birch (*Betula pubescens var pumila* (L.), but if needed, they can also consume understory species that are present in their distribution range (Ammunét et al., 2015). With changing climate conditions, we might expect more severe insect outbreaks (Post et al., 2009).

One of the few recent studies that analyzed BVOC emissions following insect herbivory in the Subarctic found that herbivory-mimicking methyl jasmonate (MeJa) application caused a four-fold increase in monoterpene emissions and a two-fold increase in sesquiterpene emissions from the dwarf birch, *B. nana*, compared to branches without MeJa application (Li et al., 2019). However, more field studies are required to quantify herbivory-BVOC emission relationships as we face potential distribution shifts in insect herbivores towards higher elevations/latitudes and increases in background herbivory, and insect outbreaks, with changing climate.

Insect herbivory can alter the proportional composition of BVOC blends emitted by plants, called chemical communication displays (CCDs) (Junker et al., 2018). CCDs are used to convey information, either in specific ratios (Bruce et al., 2005) or with specific key compounds (Sakurai et al., 2004). To assess the change in CCDs, one can calculate the phenotypic integration (PI), which is a measure of covariation patterns among complex traits, like BVOC blends (Pigliucci and Preston, 2004). A high PI reflects tightly intercorrelated traits, due to a need of specific configuration of BVOC blends for optimal performance and information transfer, whereas a low PI is suggestive of weakly intercorrelated traits (Junker et al., 2018). A recent study by Junker et al. (2018) tested the effect of herbivory on the PI of plant volatile CCDs and found that plants that were exposed to insect herbivory had less intercorrelated BVOC blends (lower PI) than control plants (higher PI).

In this work, we aimed to assess how quantitative and qualitative BVOC emissions change with increasing insect feeding intensity (low-intensity herbivory that is above background levels) in the Subarctic mountain birch forest. We conducted three independent field experiments in northern Norway and Sweden, manipulating the larval densities of geometric moths (*O. brumata* and *E. autumnata*) on *B. pubescens* var *pumila (L.)* to: (1) determine quantitative relationships between insect abundance, leaf area loss, and BVOC emission rates, (2) assess how BVOC blend composition changes with increased larval density, and (3) assess how PI of plant BVOCs changes with insect feeding.

## Materials and Methods

### Study Areas

The study was carried out in a mountain birch forest near the Abisko Scientific Research Station, Subarctic Sweden (68°21′N, 18°49′E, 385 m a.s.l.) and in Tromsø, Norway (69°38′N, 18°57′E, 50 m a.s.l.) during the growing season in 2018. The experiment was conducted in two locations during three periods: 1) June 28–July 1 in Abisko (thereafter called *Abisko-1*), 2) July 2–6 in Abisko (*Abisko-2*), and 3) June 7–12 in Tromsø. The birch forest in Abisko is characterized as heath forest dominated by mountain birch with ground vegetation dominated by *Empetrum nigrum* ssp. *hermaphroditum* Hagerup, *Vaccinium myrtillus* L., and/or *Vaccinium vitis-idaea* L. (Sonesson and Lundberg, 1974; Tenow and Bylund, 2000). The Tromsø site is located in a heath and low herb dominated mature birch forest on a south facing slope. The soil type at both sites is leptosol (Makarov et al., 2008; Jones et al., 2010).

### Experimental Design

Twenty healthy-looking branches, each from a separate mountain birch individual, were selected for each experiment. All selected branches were at about 1.5 m height from the ground. The total leaf area per branch varied between 150 and 253 cm^2^ in *Abisko-1*, 256 and 522 cm^2^ in *Abisko-2*, and 135 and 337 cm^2^ in *Tromsø*. The birch stand in Tromsø suffered from severe aphid infestation. Aphids were removed with a brush before herbivory treatments commenced.

In Abisko, a mix of larvae of the locally dominant geometrid moths, winter moth (*O. brumata*) and autumnal moth (*E. autumnata*), was used. The moth larvae were collected from a mountain birch forest close to Abisko (68°18′32.2′N, 19°12′07.3′E) and reared in the laboratory on detached birch branches until use. A mixture of larval instars was used during the experiment. Due to very low population levels of both moth species in the Tromsø area during 2018, larvae for the Tromsø experiment were collected at an outbreak site in NE Finnmark (69°41′16.0′N, 18°49′43.3′E). The larvae were all winter moth collected in their 2^*nd*^ instar, brought to Tromsø, and reared in the laboratory on detached birch branches until use. All larvae were in their 4^*th*^–5^*th*^ instar during the experiment. Both species used in the experiments are naturally outbreak species in both regions.

In each experiment, we subjected branches to different levels of insect feeding by geometrid moth larvae. Trees growing close to each other formed a block, within which they were randomly selected for the following treatments: 0, 5, 15, 30, or 50 larvae per branch (*Abisko-1* and *Abisko-2*) and 0, 5, 15, or 30 larvae per branch (*Tromsø*). Larvae were gently applied to leaves using a soft brush, and branches were enclosed in transparent mesh bags. After two (*Abisko-1* and *Abisko-2*) or four (*Tromsø*) days of insect feeding, we removed the mesh bags, larvae and their feces, and measured BVOC emissions. For each density treatment, there were four replicates for experiments in Abisko, and five replicates in Tromsø.

### BVOC Measurements

Biogenic volatile organic compounds were captured using a branch enclosure method described previously (Vedel-Petersen et al., 2015). Pre-cleaned (120°C for 1 h) polyethylene terephthalate (PET) bags (35 × 43 cm, ca. 1,300 ml) were used as branch enclosures through which air was pushed with pumps and BVOCs were trapped from outgoing air on stainless steel adsorbent cartridges (150 mg Tenax TA, 200 mg Carbograph 1TD, Markes International Limited, Llantrisant, United Kingdom).

Each PET bag was ventilated before the measurement for approximately 5 min with an inflow rate of 1,000 ml min^–1^. Subsequently, the adsorbent cartridge was inserted via a cut corner into the PET bag and secured with wire. During the 15-min sampling period, air was circulated through the enclosure with an inflow rate of 300 ml min^–1^ and an outflow rate of 200 ml min^–1^ through the adsorbent cartridge. The excess air leaked out from the opening where the branch entered the bag. The incoming air was filtered for particles and background hydrocarbons, and scrubbed for ozone, to avoid losses of highly reactive BVOCs (Valolahti et al., 2015; Kramshøj et al., 2016). After sampling, the cartridges were sealed with Teflon-coated brass caps and stored at 5°C until analysis. *In situ* blank samples were collected from empty PET bags to account for compounds derived from the sampling materials and analysis system. A new, pre-cleaned PET bag was used for each measurement.

Air temperature and relative humidity inside the enclosures were monitored during sampling with iButtons (Hygrochron, Maxim Integrated, San Jose, CA, United States) (Supplementary Table S1). Light intensity was monitored using a Photosynthetic Light (PAR) Smart Sensor (S-LIA-M003, Onset Computer Corporation, Bourne, MA, United States).

After BVOC collection, branches were cut and stored cooled. In the lab, leaves were cut from the petiole, scanned at 300 dpi using an OpticSlim 1180 scanner (Plustek, Santa Fe Springs, CA, United States). ImageJ was used to determine the leaf area of the scanned leaves as well as the leaf area eaten by larvae, which was estimated by reconstructing the damaged leaf margin using Adobe Photoshop (Adobe Photoshop 2018). In the few cases where leaf scans were not available due to low quality, the missing values were replaced with the average leaf area of the treatment. Water-saturated fresh mass was weighed and the leaves were oven dried at 70°C for 72 h to determine dry mass.

### BVOC Analysis

Biogenic volatile organic compound samples were collected in adsorbent cartridges and analyzed using gas chromatography-mass spectrometry (7890A GC coupled with a 5975C inert MSD, Agilent, Santa Clara, CA, United States) after thermal desorption at 250°C for 10 min (TD100-xr, Markes International Ltd, Llantrisant, United Kingdom). The carrier gas was helium and the oven temperature was held at 40°C for 1 min, then raised to 210°C at a rate of 5°C min^–1^, and to 250°C at a rate of 20°C min^–1^. An HP-5 capillary column (50 m length, 0.2 mm diameter, 0.33 μm film thickness, California, United States) was used for BVOC separation. See Vedel-Petersen et al. (2015) for further details on the BVOC analysis.

Chromatograms were analyzed using PARADISe v. 3.8 software (Johnsen et al., 2017). Compounds were identified using pure standards, when available (Supplementary Table S2), or tentatively identified using the Mass Spectral Library (NIST, 2014). Compounds were included in the dataset if they were present in 30% of all samples in an experiment and had a match factor (MF) above 800 when compared against the Mass Spectral Library (NIST, 2014). BVOC concentrations in blanks were subtracted from those in the samples.

BVOC emission rates (ER, ng cm^–2^ h^–1^) were expressed on a leaf area basis and calculated by:

$$\text{ER}=\frac{\left[C_{\text{out}}-C_{\text{in}}\right]\,Q}{\text{LA}}$$

where C_out_ is the concentration of BVOCs in the sampled air, C_in_ was expected to be zero as the inlet air was filtered, *Q* is the inflow rate, and LA is the leaf area of the branch (Ortega and Helmig, 2008).

BVOC concentrations were quantified using external standards (Supplementary Table S2). For quantification of compounds where pure standards were unavailable, we used *α*-pinene for monoterpenes and homoterpene (E)-4,8-dimethylnona-1,3,7-triene (DMNT), (E)-β-caryophyllene for sesquiterpenes (SQT), hexanal for green leaf volatiles (GLV), and toluene for benzenoids and other compounds. We categorized compounds into the following groups: monoterpenes (MT), homoterpene DMNT, SQT, GLV [only synthesized from lipoxygenase (LOX) pathway], benzenoids and other compounds (i.e., compounds that are not included in any of the above-mentioned groups).

### Statistical Analyses

All statistical analyses were performed within the R statistical framework, version 3.6.2 (R Core Team, 2019). To assess the overall effect of insect herbivory on the emission rates of total BVOCs and different BVOC compound groups, emission rates were the response variable and larval density was the predictor variable. We performed linear mixed-effect models (LMM), fitted with maximum likelihood (ML), using the “lme” function from the *nlme* package (Pinheiro et al., 2013). This was followed by post-hoc multiple comparisons with Tukey’s HSD using the “glht” function from the *multcomp* package (Hothorn et al., 2016). LMM were chosen to account for the randomized complete block design, in which blocks were considered as random effects, and plants were nested within blocks. For these analyses, we tested the assumptions of normality and homogeneity of variance of the BVOC data using Shapiro–Wilk normality tests and Levene’s tests, respectively. Due to the rightward skew of the BVOC data, we applied a log(*x* + 1) transformation.

We were also interested in the strength of the relationship between the leaf area eaten and emission rates of total BVOCs and for each compound group, separately. Therefore, we analyzed linear models, where leaf area eaten was the predictor variable and the emission rate was the response variable.

To test the effects of temperature and humidity on BVOC emissions, we ran multiple regression analysis and found no significant effects of air temperature and humidity on BVOC emissions across all experiments (data not shown).

To understand if and how the composition of the BVOC blend in terms of BVOC groups changed upon increased insect herbivory, we performed principal component analysis (PCA) using the “prcomp” function and *ggbiplot* package in R (Vu, 2011). Data were zero-centered and scaled to unit variance.

We used random forest (RF), a well-established machine learning algorithm, to assess whether the emission patterns of individual BVOCs could be used to classify the samples into groups and to identify the compounds responsible for the potential separation (Breiman, 2001, 2003). We divided herbivory treatments into two groups based on PCA analysis, which showed two main clusters (see section “Results”): low (<4% leaf area eaten, corresponding to low to background herbivory) and high (>4%). That gave us the following sample sizes: *n*_*low*_ = 11, *n*_*high*_ = 9 (*Abisko-1*), *n*_*low*_ = 14, *n*_*high*_ = 6 (*Abisko-2*), *n*_*low*_ = 11, *n*_*high*_ = 9 (*Tromsø*).

We used the “randomForest” function from the *randomForest* package, with herbivory treatment (low or high) as the response variable and BVOCs (proportional data) as the predictor variable. We drew *n*_*tree*_ = 100,000 bootstrap samples with *m*_*try*_ = 8 (*Abisko-1*) and m_*try*_ = 10 (*Abisko-2 and Tromsø*).

Classification of low and high insect herbivory was further used to calculate the PI of plant BVOCs for each of the experiments and for comparisons between low and high herbivory, we followed a standard method (Wagner, 1984; Herrera et al., 2002; Pérez-Barrales et al., 2007; Junker et al., 2018). We determined the Pearson’s correlation coefficient for relationships between each combination of individual compounds (emission rates in absolute amounts) and calculated eigenvalues for the resulting correlation matrix. The variance of the eigenvalues gives the integration index, a measure of the magnitude of PI. To account for the varying sample sizes between experiments, the integration index was standardized by subtracting the expected value of integration under the assumption of random covariation [random covariation = (number of substances emitted − 1)/number of samples] and then dividing by the potential maximum value of PI for the given dataset, which is equal to the number of substances emitted by plants. To test the statistical significance between low and high insect herbivory, we ran a linear model with PI as the response variable and herbivory (low or high) as the predictor (*n* = 3).

To define the covariance patterns among plant BVOCs, and how they differ between low and high insect herbivory we calculated the connectance, which is a measure of the proportion of realized connections compared to the number of possible connections between the compounds. Only correlations with a *p*-value <0.05 were included.

## Results

### Quantitative Relationships Between Insect Larval Density and BVOC Emission Rates

The percentage of leaf area eaten by larvae during two to four days of feeding, varied from 0.5 ± 0.3% (mean ± SE) in the lowest larvae group to 10 ± 3% in the highest larvae group (Table 1). Hence, our results represent low intensity herbivory, considered to be at or just above background herbivory levels. Leaf area eaten was positively correlated with the number of larvae (Supplementary Figure S1).

**TABLE 1:** BVOC emission rates (ng cm^–2^ leaf area h^–1^) of mountain birch for different larval densities. Data are presented as mean ± SE (*n* = 5 and *n* = 4 for the *Abisko* and *Tromsø* experiments, respectively). MT, monoterpenes; HT, homoterpene DMNT; SQT, sesquiterpenes; GLV, green leaf volatiles. n.d. stands for not detected.

Experiment	Larval density	Leaf area eaten (%)	Emission rates (ng cm^–2^ leaf area h^–1^)						
			MT	HT	SQT	GLV	Benzenoids	Other	Total
*Abisko-1*	0	n.d.	0.01 ± 0.002	n.d.	0.03 ± 0.01	0.1 ± 0.04	0.2 ± 0.1	0.6 ± 0.1	1 ± 0.2
	5	2 ± 0.4	0.03 ± 0.005	0.01 ± 0.004	0.02 ± 0.003	0.2 ± 0.1	0.2 ± 0.1	0.4 ± 0.1	1 ± 0.2
	15	4 ± 1	0.1 ± 0.02	0.1 ± 0.02	0.1 ± 0.01	2 ± 0.3	0.2 ± 0.1	1.5 ± 0.2	4 ± 0.4
	30	4 ± 0.5	0.2 ± 0.1	0.1 ± 0.03	0.1 ± 0.01	1.5 ± 0.3	0.2 ± 0.1	1 ± 0.2	3.1 ± 0.4
	50	7.3 ± 1.4	0.2 ± 0.1	0.1 ± 0.01	0.1 ± 0.03	3 ± 0.6	0.3 ± 0.1	1.2 ± 0.2	5 ± 1
*Abisko-2*	0	0.1 ± 0.1	0.1 ± 0.01	n.d.	0.1 ± 0.02	1 ± 0.4	0.4 ± 0.1	1.5 ± 0.4	3 ± 0.5
	5	2 ± 1	0.5 ± 0.2	0.1 ± 0.1	0.1 ± 0.04	0.3 ± 0.2	0.3 ± 0.1	2 ± 1	3.3 ± 1
	15	3 ± 1.5	2 ± 1	0.2 ± 0.04	0.2 ± 0.1	1.2 ± 0.3	0.6 ± 0.2	2.6 ± 0.5	7 ± 1.5
	30	7 ± 2.5	3.4 ± 1.4	0.5 ± 0.1	0.4 ± 0.2	1.4 ± 0.7	0.7 ± 0.2	5.2 ± 1.2	11.6 ± 3
	50	10 ± 3.4	5.3 ± 2.5	0.7 ± 0.2	0.6 ± 0.1	5 ± 3	0.8 ± 0.3	5.2 ± 1.3	18 ± 6
*Tromsø*	0	0.5 ± 0.3	0.04 ± 0.01	0.01 ± 0.01	3.3 ± 1.4	0.2 ± 0.1	0.1 ± 0.04	0.5 ± 0.1	4.2 ± 1.5
	5	2 ± 0.6	0.15 ± 0.1	0.01 ± 0.01	2.2 ± 1.1	0.5 ± 0.2	0.6 ± 0.4	0.6 ± 0.2	4.1 ± 1.3
	15	6 ± 1	0.3 ± 0.1	0.1 ± 0.02	3 ± 1	2 ± 1	1 ± 0.6	0.7 ± 0.2	6.6 ± 1.6
	30	9 ± 0.6	0.2 ± 0.04	0.1 ± 0.03	2.4 ± 1	6.3 ± 1.3	1 ± 0.8	1.5 ± 0.3	11.4 ± 2.7

**FIGURE 1:**
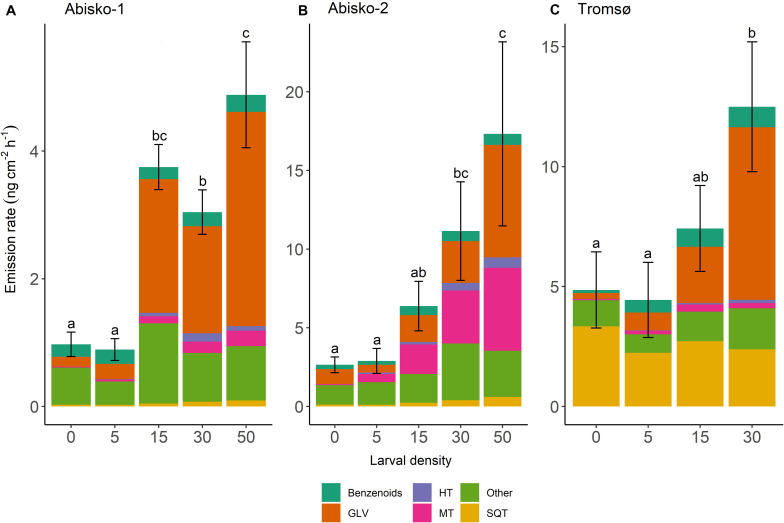
Effects of larval density on total BVOC emissions in three experiments; **(A)** *Abisko-1*, **(B)** *Abisko-2*, and **(C)** *Tromsø*. The total BVOC emissions, expressed as ng cm^–2^ leaf area h^–1^, are presented stacked for the following compound groups: Benzenoids; GLV, green leaf volatiles; HT, homoterpene DMNT; MT, monoterpenes; Other; SQT, sesquiterpenes. Larval density represents the number of larvae added per branch. The bars show mean, *n* = 4 (Abisko) and *n* = 5 (Tromsø), and the error bars show ± standard error of the total BVOC emissions. Within an experiment, bars labeled with different letters are significantly different from each other (Tukey’s post hoc test; Supplementary Table S4).

Larval density strongly affected quantitative BVOC emissions (Supplementary Table S3). On average across all experiments, the highest larval density (50 or 30 larvae/branch) caused a five-fold increase in total BVOC emissions compared to the controls with no larvae (Figure 1 and Table 1). Larval feeding increased emissions of the homoterpene DMNT (109-fold increase at the highest larval density), monoterpenes (34-fold increase), and GLVs (20-fold increase) to the greatest extent (Figure 1 and Table 1).

While the effects of larval density on BVOC emissions were mostly consistent across experiments, we observed some differences between the two locations. In Abisko, increasing larval density significantly increased emission rates of all compounds groups, except for benzenoids. In the Tromsø experiment, larval density significantly increased emissions of homoterpene DMNT and GLVs, but did not significantly increase those of monoterpenes, SQT, or benzenoids (Supplementary Table S3).

Across all experiments, feeding by five larvae per branch did not change the total BVOC emissions (Figure 1). However, when larval density increased from 15 to 30, and 50, BVOC emissions increased with increasing larval density (Supplementary Table S4). In addition, the magnitude of responses varied among BVOC groups and between experiments, and different insect larval densities were needed to cause an increase in emissions among BVOC groups. For example, in the *Abisko-1* experiment 15 larvae caused 53-fold increase in homoterpene DMNT emissions and 14-fold increase in GLVs, while monoterpenes and SQT were only significantly increased with ≥30 larvae/branch (12-fold and three-fold increases in emissions, respectively) (Table 1, Supplementary Table S4). Interestingly, larval feeding decreased sesquiterpene emissions in the *Tromsø* experiment by 29% when branches were exposed to 30 insects compared to controls (Table 1).

### BVOC Composition Change Upon Increased Larval Density

**FIGURE 2:**
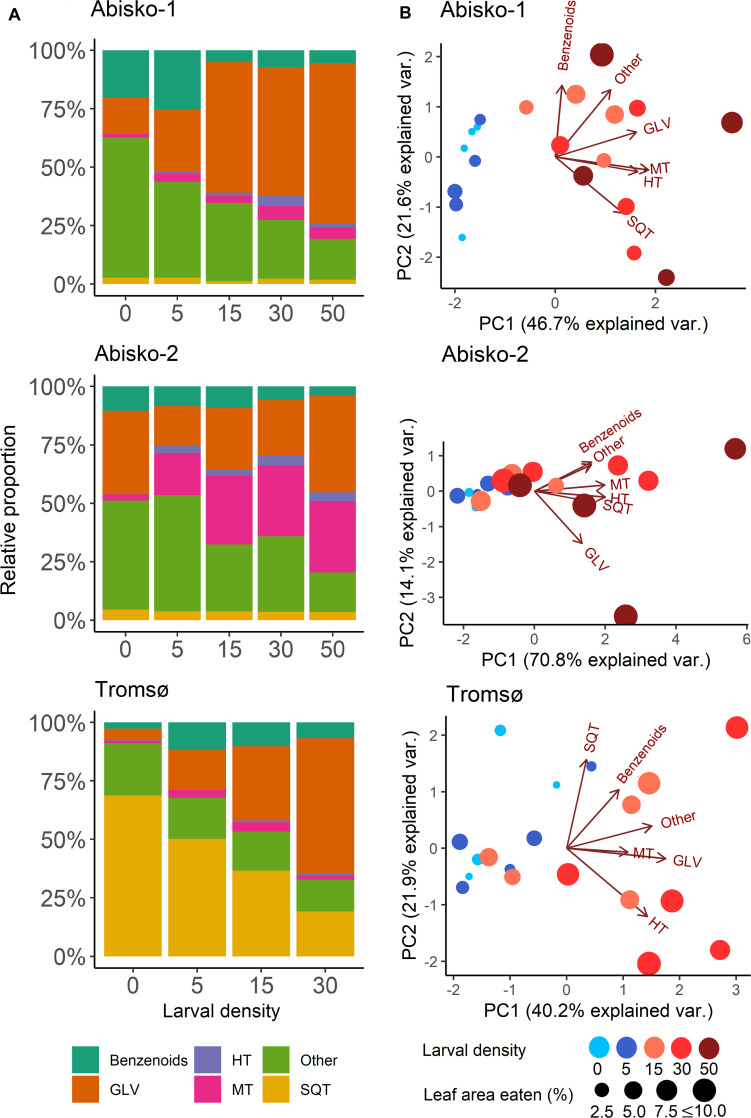
Effects of increased larval density (0, 5, 15, 30 or 50 larvae per branch) on BVOC blends in experiments *Abisko-1*, *Abisko-2*, and *Tromsø*. **(A)** Changes in relative proportions of different BVOC groups: Benzenoids; GLV, green leaf volatiles; HT, homoterpene DMNT; MT, monoterpenes; Other; SQT, sesquiterpenes. **(B)** Principal component analysis biplots on BVOC group composition. The score values for the measured branches are shown colored for different larval densities and sized based on the leaf area eaten. Loadings for each BVOC group are presented with arrows. The variance explained by each principal component (PC) is shown in parentheses.

Increased larval density also altered the BVOC blend composition in all three experiments (Figure 2).

Overall, the first principal component of the PCA (explained variances ranging from 40% for *Tromsø* data to 71% for the *Abisko-2* experiment) clearly separated the samples with higher leaf level damage and thus, higher larval densities, from the control (Figure 2B). However, separation between the control and the lower larval densities (five larvae per branch) was unclear. The relative proportions of different BVOC groups changed with increasing larval density (Figure 2A). Across all experiments, the proportion of homoterpene DMNT increased with increasing larval density and the relative proportion of GLVs showed clear increases in the *Abisko-1* and *Tromsø* experiments, as also demonstrated by the PCA loadings plot (Figure 2B). The relative proportion of monoterpenes increased for the Abisko experiments, and was not clearly altered for the *Tromsø* experiment (Figure 2A, also note the low loading value in Figure 2B). For SQT, the relative proportion decreased in the *Tromsø* experiment, and was unaffected in the Abisko experiments (Figure 2A).

In all experiments, a large fraction of the total BVOCs consisted of non-terpenoid BVOCs (Figure 2A). In the *Abisko* experiments, other BVOCs contributed 17–60% to the total BVOCs, whereas in the *Tromsø* experiment, other BVOCs contributed only 14–22% to the total BVOCs. Across all experiments, benzenoids contributed 3–25% to the total BVOCs.

### Relationships Between Leaf Area Eaten and BVOC Emissions

**FIGURE 3:**
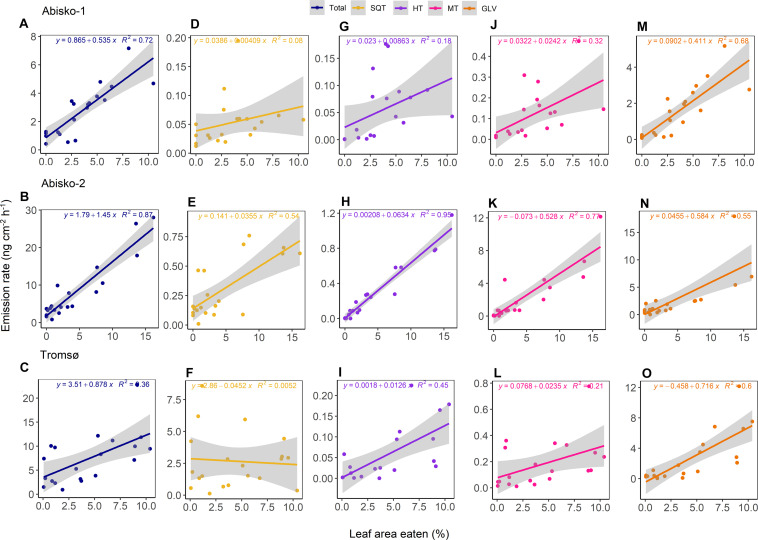
The relationship between the leaf area eaten and BVOC emission rates for *Abisko-1*, *Abisko-2*, and *Tromsø* experiments. Scatter plots and linear fits are shown for total BVOCs **(A–C)** and the compound groups: SQT, sesquiterpenes **(D–F)**; HT, homoterpene DMNT **(G–I)**; MT, monoterpenes **(J–L)**; GLV, green leaf volatiles **(M–O)**. Emission rates are expressed as ng cm^–2^ leaf area h^–1^ and leaf area eaten is expressed as percentage. For each of the experiments, *n* = 20.

The emission rates of total BVOCs increased with increasing leaf area eaten with slopes ranging from 0.5 ± 0.1 ng cm^–2^ h^–1^ to 1.5 ± 0.1 ng cm^–2^ h^–1^ across all experiments (Figures 3A–C). Of the individual compound groups, homoterpene DMNT, monoterpenes, and GLV emissions were consistently positively correlated with leaf area eaten, across all experiments (Figure 3 and Supplementary Table S5). For example, the slopes of GLV emissions against leaf area eaten ranged from 0.4 ± 0.1 ng cm^–2^ h^–1^ in *Abisko-1* to 0.7 ± 0.1 ng cm^–2^ h^–1^ in *Tromsø*. For other compound groups, differences were observed between experiments. Although not statistically significant, sesquiterpene emissions increased with leaf area eaten in the Abisko experiments (Figures 3D,E), but less clear or no trends were observed in *Tromsø* (Figure 3F).

The most abundant GLV emitted across all experiments was (E)-2-hexenal, whose emissions were positively correlated with leaf area eaten; the slopes among experiments for (E)-2-hexenal ranged from 0.2 ± 0.1 ng cm^–2^ h^–1^ to 0.3 ± 0.1 ng cm^–2^ h^–1^ (Supplementary Table S6). A consistent increase in emissions with leaf area eaten was also observed for the homoterpene DMNT, which was identified based on its relative retention time match with a known standard (Supplementary Table S6). The most abundant monoterpene in the *Abisko-1* and *Tromsø* experiments was linalool, while in the *Abisko-2* experiment, it was (E)-β-ocimene (Supplementary Table S6). The most abundant sesquiterpene for *Abisko-1* and *Abisko-2* was (Z)-β-farnesene and for *Tromsø*, (E)-β-caryophyllene.

### Change of the BVOC Blend and Phenotypic Integration

Random forest correctly assigned most samples to their predefined groups [*Abisko-1* (Out-of-bag (OOB) error = 20%; low, class error = 18%; high, class error = 22%), *Abisko-2* (OBB = 5%; low, class error = 0%; high, class error = 16%) and *Tromsø* (OBB = 25%; low, class error = 18%; high, class error = 33%)] (Supplementary Figure S2). RF variable importance analysis suggested that different volatile compounds were primarily responsible for distinguishing between the groups in each experiment (Supplementary Tables S7–S9).

The average PI value across all experiments was 11 ± 1 for low insect herbivory and 6 ± 1 for high insect herbivory. PI was different between high and low insect herbivory (*F*_1,4_ = 7.8, *P* = 0.048); PI values decreased when plants were exposed to high insect herbivory for each of the three experiments (Table 2). The connectedness, which is a measure of the proportion of pairs of volatiles that significantly covaried, also decreased when plants were exposed to high insect herbivory (Table 2).

**TABLE 2:** Phenotypic integration, leaf area eaten (%), BVOC emission rates (ng cm^–2^ leaf area h^–1^), and connectedness across all experiments (*Abisko-1*, *Abisko-2*, and *Tromsø*) for low and high insect herbivory. Data are presented as mean ± SE.

		Experiment		
Variable	Group	*Abisko-1*	*Abisko-2*	*Tromsø*
Leaf area eaten	Low	1 ± 0.4	1 ± 0.4	2 ± 0.4
	High	6 ± 1	11 ± 2	8 ± 1
BVOC emission rates	Low	2 ± 0.3	4 ± 1	5 ± 1
	High	4 ± 0.4	18 ± 3	11 ± 2
Phenotypic integration	Low	13	9	10
	High	4	7	8
Connectedness	Low	0.3	0.2	0.2
	High	0.1	0.1	0.2

## Discussion

Our study shows that short-term insect herbivory significantly increased BVOC emissions from the branches damaged by larval feeding. We observed that insect herbivory, as measured by leaf area eaten (up to 10% of leaf area loss), increased BVOC emissions in Subarctic mountain birch at relatively low larval densities, for both total BVOCs and separate compound groups. Larval density strongly affected BVOC emissions. On average across all experiments, the highest larval density caused a five-fold increase in total BVOC emissions compared to the controls with no larvae. Larval density also strongly increased the emissions of homoterpene DMNT (109-fold increase at the highest larval density), monoterpenes (34-fold increase), and GLVs (20-fold increase). We found that insect herbivory changed the blend composition of BVOCs, with damaged plants producing less intercorrelated BVOC blends than undamaged ones.

Emission rates of total BVOCs and all compound groups, except for SQT in the *Tromsø* experiment, were positively correlated with the leaf area eaten observed in our experiments. Among individual compounds, we observed induction of the emissions of the homoterpene DMNT and products of the lipoxygenase pathway (LOX products, or GLVs), particularly (E)-2-hexenal, which is commonly emitted after insect herbivore feeding and is suggested to be involved in plant-to-plant signaling (Kessler and Baldwin, 2002). DMNT is usually synthesized *de novo* in response to herbivore attack (Kessler and Baldwin, 2002) and plays a role in attracting enemies of the herbivores (McCormick et al., 2019). We also found other volatiles associated with the attraction of herbivore enemies, such as the monoterpenes, linalool and (E)-β-ocimene (Clavijo McCormick et al., 2012), whose emissions also increased with increasing insect herbivory.

Earlier studies have demonstrated that SQT are released following herbivory and subsequently involved in indirect plant defenses, particularly under controlled conditions (Schaub et al., 2010; Copolovici et al., 2011; Clavijo McCormick et al., 2012). We found that sesquiterpene emissions increased with leaf area eaten in two of the three experiments (*Abisko-1* and *Abisko-2*). Interestingly, insect feeding did not affect sesquiterpene emissions, nor the emission of (E)-β-caryophyllene, which was the most abundant sesquiterpene in the *Tromsø* experiment. This discrepancy could have several explanations. First, confounding effects from the severe aphid infestation, which had naturally occurred in the Tromsø experimental area prior to our experiment, could have influenced sesquiterpene emissions. It is known that attack by phloem-feeding insects such as aphids often triggers the salicylic acid pathway, which can antagonize or neutralize the jasmonic acid-mediated defense responses activated by tissue-chewing insects (Dicke et al., 2009; Schweiger et al., 2014). For instance, simultaneous feeding on cotton plants (*Gossypium hirsutum* L.) by phloem-feeding whiteflies (*Bemisia tabaci*) and tissue-chewing caterpillars (*Spodoptera exigua*) reduced BVOC emissions as compared to feeding by only caterpillars (Rodriguez-Saona et al., 2003). Second, the observed differences in BVOC responses to herbivory between geographical locations of the experiment could also be due to the inherent differences in abiotic and biotic environments between the locations, such as different insect outbreak histories or genetic differences (López-Goldar et al., 2019; McCormick et al., 2019). For example, the two locations were at different phases of the geometrid moth outbreak cycle: In the Abisko area, the latest moth population peak was in 2012 (Olofsson et al., 2013), whereas in the Tromsø area, it was in 2014 (Vindstad et al., 2019, see also www.coat.no/en/Tundra-forest-ecotone). In addition, the genetic differences among and even within the populations are known to affect the BVOCs emitted by plants (Bäck et al., 2012; López-Goldar et al., 2019). For example, López-Goldar et al. (2019) found substantial variations in sesquiterpene production among populations of maritime pine (*Pinus pinaster* Ait.) along a large geographic gradient. On the other hand, it could also be due to differences in the experimental setup, because we used a mixture of *E. autumnata* and *O. brumata* in the Abisko experiments, while only *O. brumata* was used in our *Tromsø* experiment.

Under natural conditions, plants are often subjected to multiple stress factors (Holopainen and Gershenzon, 2010). We found that control branches emitted the typically stress-induced GLVs at low rates and further, that treatment with five larvae feeding on plants for two to four days did not significantly alter the BVOC emission rates or blend compositions compared to the control branches. This result could have several explanations. First, the lack of differences between the control and branches with five insects added could be a result of a long course of interactions via cyclic outbreaks of geometrid moths, which are common in the mountain birch forests (Bylund, 1999; Jepsen et al., 2011, 2013). Second, earlier feeding by herbivores on the target branches prior to the start of the treatment could have interfered with our treatments. Third, it could be that control branches or other parts of the trees were experiencing damage by other herbivores and that could cause a systemic response in the control branches (Hilker and Schmülling, 2019). McCormick et al. (2019), who assessed herbivore-induced BVOCs under field conditions, argued that laboratory studies conducted under well-controlled conditions show contrasting results to field studies. It suggests that confounding factors are experienced in the field and therefore, to better understand the ecological relevance of herbivore-induced BVOC emissions, more experiments should be conducted in as near-natural conditions as possible.

Insect herbivory altered the composition of BVOCs. Plants that had feeding damage above background herbivory levels, produced less integrated BVOC blends compared to plants that had damage below background herbivory levels. This finding is consistent with that of Junker et al. (2018), who argued that herbivory affects the covariance patterns via plants inducing individual BVOCs to attract herbivore predators or to prevent subsequent herbivore attacks. In contrast, in plants that have not been exposed to herbivores, the quantities of BVOCs are largely regulated by their flux through specific pathways. Thus, several compounds from the same pathway would be similarly affected. Our RF analysis indicated that certain GLVs, monoterpenes, and SQT were important for distinguishing the BVOC blends from low insect herbivory and high insect herbivory, although these compounds were experiment-specific. Changes in covariance patterns during insect herbivore attacks, such as insect outbreaks, could potentially disrupt the volatile cues herbivores are using to locate their host plants (Bruce and Pickett, 2011).

Induced BVOCs contribute a substantial fraction to the total BVOC emissions from plants (Arneth and Niinemets, 2010; Faiola and Taipale, 2020; Noe and Niinemets, 2020). Nevertheless, there is no explicit consideration of herbivory-induced emissions in the currently used mainstream BVOC models, e.g., MEGAN (Guenther et al., 2012) or LPJ-GUESS (Smith, 2001). The herbivory-related compounds quantified in this study can be used to separate herbivory-related stress compounds from other stresses, such as storm, cold temperature, and drought (Guenther et al., 2012). The significant relationships we found between leaf area eaten and emission rates reflect background herbivory levels but can potentially be extrapolated to larger regions and longer time periods. For example, leaf area eaten could be quantified from the reductions in remote sensing-observed leaf area index (LAI), e.g., using normalized difference vegetation index (NDVI) (Jepsen et al., 2009; Olsson et al., 2016). In this way, the relative contributions of induced and constitutive emissions to total BVOC emissions, as well as their feedbacks to atmospheric chemistry, can be estimated. The current study was not specifically designed to evaluate scenarios mimicking insect outbreaks, but rather low intensity herbivory, that is above the background levels. However, a comprehensive estimation of herbivory impacts on BVOC emissions would need to also account for impacts of higher insect densities present during insect outbreaks as well as the responses after the outbreak is finished and the trees are completely defoliated. In some cases the complete defoliation is followed by a second flush of leaves. In other, the trees do not recover. Both background herbivory levels and insect outbreaks are predicted to increase with changing climate, and therefore, experiments using the same experimental set up but higher insect densities would further improve our understanding of plant responses to more severe insect pressure.

To conclude, we showed that short term, low intensity insect herbivory increased BVOC emissions from the branches damaged by larval feeding in Subarctic mountain birch forests. These results provide a much-needed, quantitative understanding of the relationships between the severity of insect herbivore damage, at leaf area losses corresponding to above background herbivory levels, and emissions of both constitutive and induced BVOCs. To address the ecological relevance of the BVOC blends altered by herbivory, we assessed the changes in PI of BVOC blends and found that the damaged plants produced less intercorrelated BVOC blends than the undamaged ones. Our results have important and practical implications for modeling the relative contributions of induced and constitutive emissions to the total emissions, as well as their feedbacks to atmospheric chemistry. We propose further research to assess the BVOC responses to severe insect pressure (e.g., “outbreak” densities) on plants in as near-natural conditions as possible, using a similar experimental design to better understand how biotic stress and changing climate affect BVOC emissions and plant–insect interactions.

## Data Availability Statement

The data of this study are available upon request from the corresponding author.

## Author Contributions

TL and RR designed the experiments. JJ provided the study site and insects for the experiment in Tromsø. JR, TL, and IR collected data. JR performed data analysis with contributions from TL and RJ. RR contributed with resources. JR wrote the manuscript and all authors contributed to the article and approved the submitted version.

## Conflict of Interest

The authors declare that the research was conducted in the absence of any commercial or financial relationships that could be construed as a potential conflict of interest.
