# Supplementary material for: Insect Herbivory Strongly Modifies Mountain Birch Volatile Emissions
Source: Front Plant Sci. 2020 Oct 27;11:558979. doi: 10.3389/fpls.2020.558979 (PMC7652793; doi:10.3389/fpls.2020.558979)
Supplement: Supplementary file 1 [file Data_Sheet_1.docx]

Supplementary Figures

**
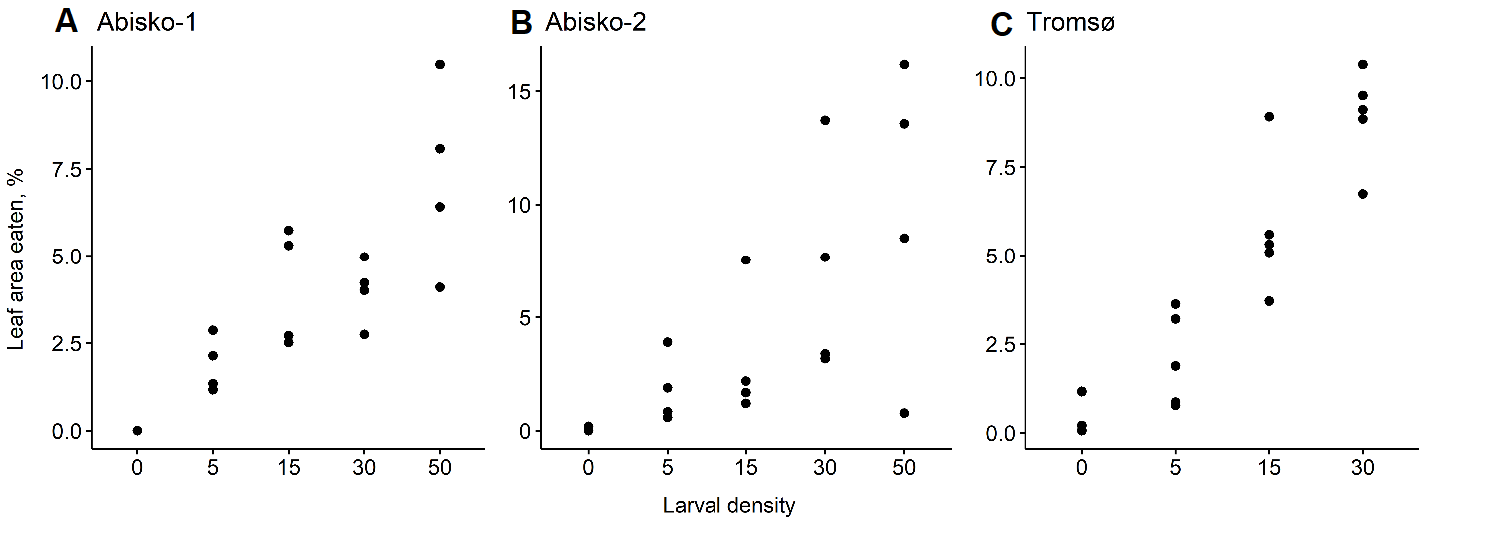
**

**Figure S1** The relationship between the numbers of larvae added and leaf area eaten for three experiments: **(A)** *Abisko-1*, **(B)** *Abisko-2* and **(C)** *Tromsø*. The leaf area eaten (%) were positively correlated with the insect density across all experiments (*Abisko-1*: Pearson correlation coefficient: r^2^ = 0.83, P < 0.001, CI [0.81 to 0.85]; *Abisko-2*: Pearson correlation coefficient: r^2^ = 0.71, P < 0.0001, CI [0.68 to 0.73]; *Tromsø*: Pearson correlation coefficient: r^2^ = 0.93, P < 0.0001, CI [0.92 to 0.93]. High variation and lower leaf area eaten for some replicates in *Abisko-1* with 30 insects and in *Abisko-2* with 30 and 50 insects was observed due to high larvae mortality in mesh bags during the larvae feeding period.

**
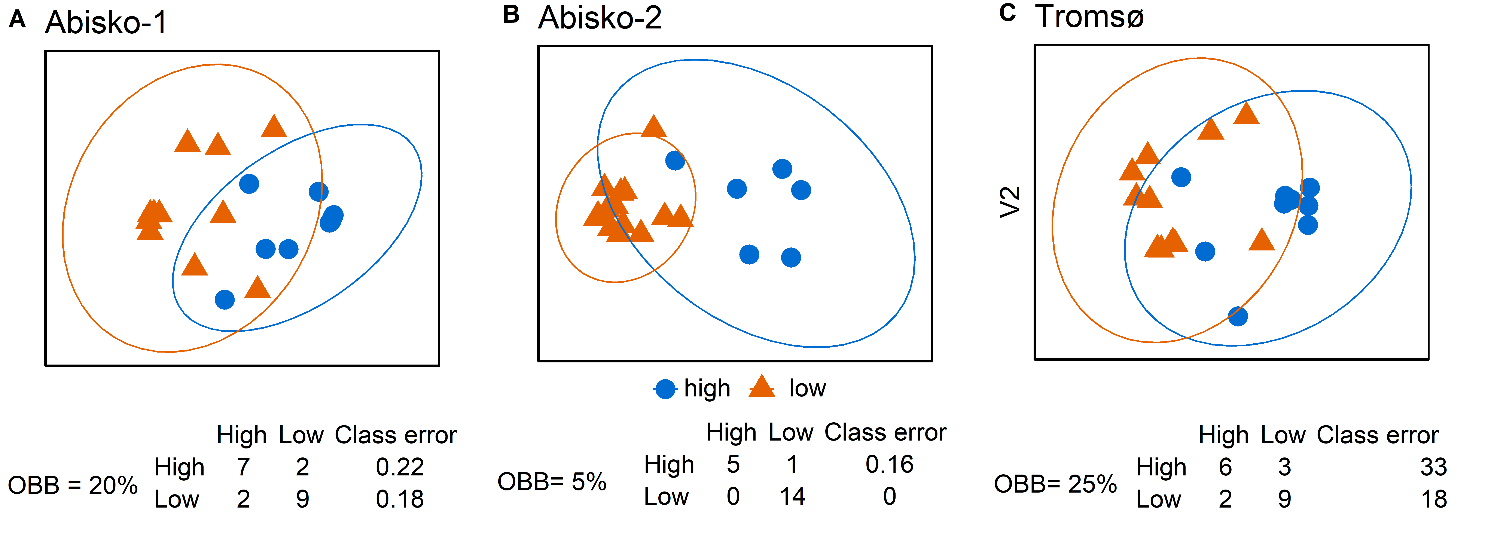
Figure S2** Multidimensional scaling (MDS) plots of the results of Random Forest analysis for separating samples into low and high insect herbivory for each of the three experiments: **(A)** *Abisko-1*, **(B)** *Abisko-2* and **(C)** *Tromsø*. MDS plot is drawn using Random Forests´ proximities and gives two clusters (low and high insect herbivory). Each graph also consists of error matrix that shows the total out-of-bag error (OBB) and class error for each of the groups separately.
